# *catena*-Poly[[aqua­zinc(II)]-μ-*N*,*N*′-bis­(2-cyano-3-eth­oxy-3-oxoprop-1-en­yl)benzene-1,2-diaminido]

**DOI:** 10.1107/S1600536814008381

**Published:** 2014-04-18

**Authors:** Monica Fuchs, Thomas Zevaco, Eckhard Dinjus, Olaf Walter

**Affiliations:** a IKFT, KIT-Campus Nord, Hermann-von-Helmholtz-Platz 1, 76344 Eggenstein-Leopoldshafen, Germany

## Abstract

The slightly yellow-coloured title complex, [Zn(C_18_H_16_N_4_O_4_)(H_2_O)]_*n*_, crystallizes with one mol­ecule in the asymmetric unit. The structure clearly shows the *mer*-η^4^ *O*,*O*,*N*,*N*-binding mode of the *N*,*N*′-bis-(2-cyano-ethyl­propeno­yl)-1,2-di­amido­benzene ligand stabilizing the Zn centre of a distorted octa­hedral environment. The fifth coordination site in one apical position is held by a coordinating solvent water mol­ecule whereas the complete octa­hedral coordination sphere is completed by coordination of one N atom from a CN group of a neighbouring mol­ecule, leading to the final polymeric structure consisting of zigzag staggered chains in parallel orientation along the *c*-axis direction. Between the coord­in­ated water solvent molecule and the N atoms of uncoord­in­ated cyano-groups of neighboured units, two H-bridge bonds are formed. One of these H-bridge bonds is of inter- whereas the other of intra-strand nature, leading to a two-dimensional network parallel to (110) stabilizing the supramolecular structure. Six Zn—O or Zn—N bonds are found with lengths ranging from 2.061 (1) to 2.185 (1) Å and bond angles about the Zn atom are clustered in the ranges 79.83 (4)–104.21 (4) and 167.05 (4)–170.28 (4)°.

## Related literature   

The structures of Zn^II^ complexes with ligands stabilizing comparable complex geometries can be found in Barnard *et al.* (2009 ▶), Ryu *et al.* (2003 ▶) or Tanase *et al.* (2001 ▶). In Tanase *et al.* (2001 ▶), the ligands show comparable *N*,*N*,*O*,*O*-coordination with respect to a different ligand backbone whereas in Ryu *et al.* (2003 ▶) and Barnard *et al.* (2009 ▶), the ligands with *N*,*N*,*N*,*N*-coordination are di­amino­benzene derivatives. In Fuchs *et al.* (2014 ▶), a mononuclear Zn complex is presented with the same ligand but a dmso mol­ecule in the coordination sphere of the metal stabilizing a different complex geometry. For the synthesis, see: Jäger *et al.* (1985 ▶).
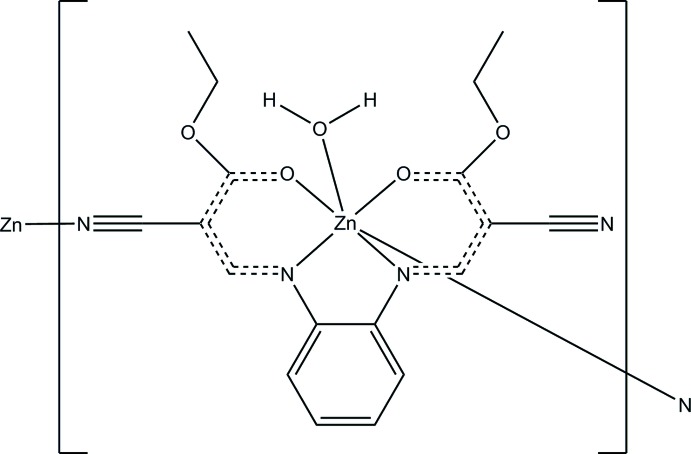


## Experimental   

### 

#### Crystal data  

[Zn(C_18_H_16_N_4_O_4_)(H_2_O)]
*M* *_r_* = 435.73
Orthorhombic, 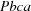

*a* = 13.9312 (11) Å
*b* = 9.2315 (7) Å
*c* = 27.423 (2) Å
*V* = 3526.7 (5) Å^3^
*Z* = 8
Mo *K*α radiation
μ = 1.43 mm^−1^
*T* = 100 K
0.10 × 0.09 × 0.07 mm

#### Data collection  

Bruker APEXII Quazar diffractometer
Absorption correction: multi-scan (*SADABS*; Bruker, 2007 ▶) *T*_min_ = 0.928, *T*_max_ = 0.953
60234 measured reflections
4294 independent reflections
3812 reflections with *I* > 2σ(*I*)
*R*_int_ = 0.025

#### Refinement  

*R*[*F* ^2^ > 2σ(*F* ^2^)] = 0.023
*wR*(*F* ^2^) = 0.063
*S* = 1.04
4294 reflections
268 parameters
H atoms treated by a mixture of independent and constrained refinement
Δρ_max_ = 0.42 e Å^−3^
Δρ_min_ = −0.43 e Å^−3^

### 

Data collection: *APEX2* (Bruker, 2007 ▶); cell refinement: *SAINT* (Bruker, 2007 ▶); data reduction: *SAINT*; program(s) used to solve structure: *SHELXS97* (Sheldrick, 2008 ▶); program(s) used to refine structure: *SHELXL2013* (Sheldrick, 2008 ▶); molecular graphics: *ORTEP-3 for Windows* (Farrugia, 2012 ▶); software used to prepare material for publication: *publCIF* (Westrip, 2010 ▶).

## Supplementary Material

Crystal structure: contains datablock(s) I. DOI: 10.1107/S1600536814008381/nk2220sup1.cif

Structure factors: contains datablock(s) I. DOI: 10.1107/S1600536814008381/nk2220Isup2.hkl

Supporting information file. DOI: 10.1107/S1600536814008381/nk2220Isup3.cdx

CCDC reference: 992382

Additional supporting information:  crystallographic information; 3D view; checkCIF report

## Figures and Tables

**Table 1:** Hydrogen-bond geometry (Å, °)

*D*—H⋯*A*	*D*—H	H⋯*A*	*D*⋯*A*	*D*—H⋯*A*
O5—H51⋯N4^i^	0.79 (2)	2.21 (2)	2.9823 (17)	168 (2)
O5—H52⋯N4^ii^	0.83 (2)	2.12 (2)	2.9405 (16)	174 (2)

Symmetry codes: (i) 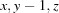; (ii) 
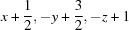
.

**Fig. 1.**
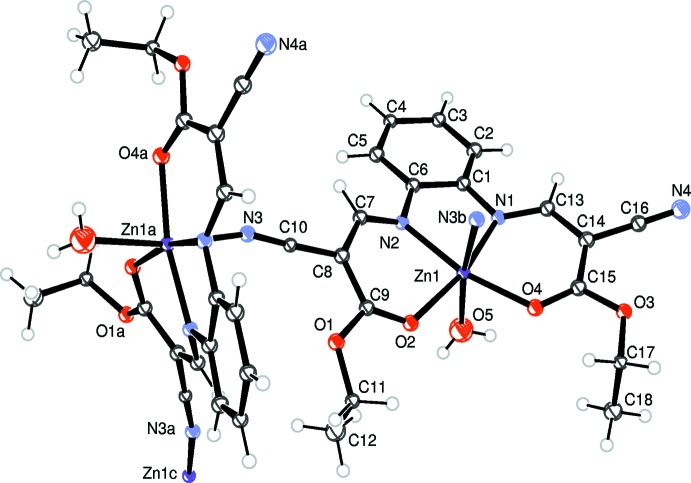
View to the a part of the polymeric chain in the molecular structure of catena-Poly-{aqua-η4-N,N,O,O- µ-N'-cyano-[N,N'-bis-(2-cyano-ethylpropenoyl)- 1,2-diamidobenzene]zinc(II)}; ellipsoids at 50% probability level (Symmetry codes for atoms with suffix a: -x, -0.5 + y, 0.5 - z, -z + 1; with suffix b: -x, 0.5 + y, 0.5 - z; and with suffix c: x, y - 1, z. For the residues with suffix b, c there is only one atom of the unit represented in the drawing).

## supplementary crystallographic information

### 1. Comment

The polymeric chain structure of title compound can be described by single units of to a Zn-centre *mer*-η^4^-O,*O*-N,*N*-coordinated *N*,*N*'-bis-(2-cyano-ethylpropenoyl)-1,2-diamidobenzene ligand with a water solvent molecule in one apical position over the coordination plane. The polymeric structure and completeness of the coordination sphere is obtained by intermolecular coordination of a N-atom of a neighboured molecule finally forming the polymeric catena-structure with Zn-atoms in distorted octahedral geometry. The Zn-O bond distances involving the ligand are accordingly determined to 2.061 (1) and 2.111 (1) Å and agree with the corresponding Zn-N bond lengths of 2.063 (1) and 2.075 (1) Å. The Zn-O bond distance to the coordinated water solvent molecule is elongated to 2.185 (1) Å but still shorter than the one to the N-atom of the cyano-group of a neighbour molecule (2.232 (1) Å) which finally leads to the formation of the polymeric catena structure. The four donor atoms of the ligand in its mer-coordination form a coordination plane, they deviate from this by in the mean 0.09 Å so that the position of the central Zn-atom with a deviation from this plane of 0.04 Å can be regarded as placed well within this plane. These findings are in agreement with its embedding in the centre of a distorted octahedron. With respect to the flexibility of Zn(ii) in the formation of different complex geometries the complex fits within Fuchs *et al.* (2014), Barnard *et al.* (2009), Ryu *et al.* (2003), or Tanase *et al.* (2001), even if for the latter case larger structural deviations are observed due to larger structural differences in the ligand constitution.

### 2. Experimental

*N*,*N*'-Bis(2-cyano-2-ethoxycarbonylethenyl)benzene-1,2-diamine was synthesized from 1,2-diaminobenzene and 2-cyano-3-methoxypropanoic acid ethyl ester according to Jäger *et al.* (1985). Under an argon atmosphere 5.00 g (14.1 mmol) *N*,*N*'-bis(2-cyanoethylpropenoyl)-1,2-diaminobenzene is suspended in 75 ml thf. 14.1 ml diethyl zinc solution (1*M* in hexane, 14.1 mmol) is added under stirring. The reaction mixture is stirred overnight, the solvent is removed under reduced pressure leaving 2 as a deep yellow solid (5.88 g, 14.1 mmol, 99.8%). Single crystals are obtained by re-crystallization from dimethylsulfoxide in an open laboratory beaker glass.

### 3. Refinement

The positions of the H atoms are calculated on geometrical positions according to the hybridization of the atoms they are bound to. The isotropic U values of these hydrogen atoms are refined in groups with respect to the hybridization of the atoms where they are bound to. A riding model was applied for the refinement of the H-atoms of the methyl-groups. The positions of H51 and H52 are located in the fourier map and refined together with their isotropic displacement parameter leading to O—H distances of 0.785 (24) and 0.826 (24) Å and a bond angle H—O—H of 111 (2)°.

### Crystal data

**Table:**

[Zn(C_18_H_16_N_4_O_4_)(H_2_O)]	*D*_x_ = 1.641 Mg m^−^^3^
*M**_r_* = 435.73	Mo *K*α radiation, λ = 0.71073 Å
Orthorhombic, *P**b**c**a*	Cell parameters from 9786 reflections
*a* = 13.9312 (11) Å	θ = 5.5–56.7°
*b* = 9.2315 (7) Å	µ = 1.43 mm^−^^1^
*c* = 27.423 (2) Å	*T* = 100 K
*V* = 3526.7 (5) Å^3^	Quader, light yellow
*Z* = 8	0.10 × 0.09 × 0.07 mm
*F*(000) = 1792	

### Data collection

**Table:**

Bruker APEXII Quazar diffractometer	4294 independent reflections
Radiation source: microfocus sealed Iµs tube	3812 reflections with *I* > 2σ(*I*)
Detector resolution: 66 pixels mm^-1^	*R*_int_ = 0.025
combined φ– and ω–scans	θ_max_ = 28.6°, θ_min_ = 1.5°
Absorption correction: multi-scan (*SADABS*; Bruker, 2007)	*h* = −18→18
*T*_min_ = 0.928, *T*_max_ = 0.953	*k* = −11→12
60234 measured reflections	*l* = −36→36

### Refinement

**Table:**

Refinement on *F*^2^	Primary atom site location: structure-invariant direct methods
Least-squares matrix: full	Secondary atom site location: difference Fourier map
*R*[*F*^2^ > 2σ(*F*^2^)] = 0.023	Hydrogen site location: inferred from neighbouring sites
*wR*(*F*^2^) = 0.063	H atoms treated by a mixture of independent and constrained refinement
*S* = 1.04	*w* = 1/[σ^2^(*F*_o_^2^) + (0.0305*P*)^2^ + 2.5256*P*] where *P* = (*F*_o_^2^ + 2*F*_c_^2^)/3
4294 reflections	(Δ/σ)_max_ = 0.001
268 parameters	Δρ_max_ = 0.42 e Å^−^^3^
0 restraints	Δρ_min_ = −0.43 e Å^−^^3^

### Special details

**Table:**

Experimental. Spectroscopic data: ^1^H NMR (400 MHz, dmso-d_6_): δ = 7.56, dd, ^3^J_HH_= 6.1 Hz, ^4^J_HH_= 3.1 Hz, 2H, H(arom); 7.05, dd, ^3^J_HH_= 6.1 Hz, ^4^J_HH_= 3.1 Hz, 2H, H(arom); 4.25, q, ^3^J_HH_= 7.1 Hz, 4H, OCH_2_; 1.29, t, ^3^J_HH_= 7.1 Hz, 3H, CH_3_. ^13^C NMR (100 MHz, dmso-d_6_): δ = 177.77; 156.27; 138.01; 124.39; 121.19; 114.89; 68.05; 60.24; 14.13. ESI-MS (m/*z*, %): 417 (100) [*M*]^+^, 418 (23) [*M*+H]^+^, 835 (15) [2*M*+H]^+^. IR (KBr, cm^-1^): 2203; 1625; 1023; 748. UV/VIS (CH_2_Cl_2_): λmax (ε) (nm, mol^-1^dm^3^cm^-1^): 241 (16522),
Geometry. All e.s.d.'s (except the e.s.d. in the dihedral angle between two l.s. planes) are estimated using the full covariance matrix. The cell e.s.d.'s are taken into account individually in the estimation of e.s.d.'s in distances, angles and torsion angles; correlations between e.s.d.'s in cell parameters are only used when they are defined by crystal symmetry. An approximate (isotropic) treatment of cell e.s.d.'s is used for estimating e.s.d.'s involving l.s. planes.

### Fractional atomic coordinates and isotropic or equivalent isotropic displacement parameters (Å^2^)

**Table:**

	*x*	*y*	*z*	*U*_iso_*/*U*_eq_	
Zn1	0.50231 (2)	0.19828 (2)	0.37062 (2)	0.00984 (6)	
O1	0.36966 (7)	−0.12202 (11)	0.28089 (3)	0.0161 (2)	
O2	0.41756 (7)	0.03163 (10)	0.34019 (3)	0.01339 (19)	
O3	0.34564 (7)	0.50414 (11)	0.45261 (4)	0.0178 (2)	
O4	0.40466 (7)	0.31264 (10)	0.41189 (3)	0.01378 (19)	
O5	0.51731 (9)	0.06806 (12)	0.43673 (4)	0.0195 (2)	
H51	0.5088 (13)	−0.014 (3)	0.4428 (7)	0.026 (5)*	
H52	0.5083 (14)	0.119 (3)	0.4611 (9)	0.035 (6)*	
N1	0.61097 (8)	0.33622 (12)	0.39350 (4)	0.0107 (2)	
N2	0.61290 (8)	0.12889 (12)	0.32640 (4)	0.0105 (2)	
N3	0.53603 (9)	−0.15532 (13)	0.19114 (4)	0.0150 (2)	
N4	0.50550 (9)	0.76393 (14)	0.47291 (4)	0.0168 (2)	
C1	0.70235 (10)	0.29789 (13)	0.37492 (4)	0.0104 (2)	
C2	0.79016 (10)	0.35644 (14)	0.38978 (5)	0.0127 (2)	
H2	0.7915	0.4271	0.4150	0.017 (2)*	
C3	0.87537 (10)	0.31270 (14)	0.36822 (5)	0.0131 (3)	
H3	0.9342	0.3561	0.3779	0.017 (2)*	
C4	0.87557 (10)	0.20536 (14)	0.33235 (5)	0.0133 (3)	
H4	0.9342	0.1760	0.3177	0.017 (2)*	
C5	0.78987 (10)	0.14213 (14)	0.31837 (5)	0.0129 (3)	
H5	0.7900	0.0664	0.2949	0.017 (2)*	
C6	0.70280 (10)	0.18889 (14)	0.33858 (4)	0.0106 (2)	
C7	0.60147 (10)	0.06126 (13)	0.28535 (4)	0.0109 (2)	
H7	0.6540	0.0611	0.2633	0.011 (3)*	
C8	0.51716 (10)	−0.01222 (15)	0.27076 (5)	0.0117 (2)	
C9	0.43345 (10)	−0.02889 (14)	0.30044 (4)	0.0116 (2)	
C10	0.52453 (9)	−0.09216 (15)	0.22679 (5)	0.0117 (2)	
C11	0.29041 (10)	−0.17030 (16)	0.31142 (5)	0.0173 (3)	
H11A	0.2381	−0.2080	0.2906	0.027 (2)*	
H11B	0.2650	−0.0872	0.3303	0.027 (2)*	
C12	0.32314 (12)	−0.28751 (16)	0.34613 (6)	0.0219 (3)	
H12A	0.3535	−0.3660	0.3276	0.027 (2)*	
H12B	0.2677	−0.3256	0.3640	0.027 (2)*	
H12C	0.3695	−0.2470	0.3693	0.027 (2)*	
C13	0.59711 (10)	0.46053 (14)	0.41516 (4)	0.0111 (2)	
H13	0.6510	0.5226	0.4188	0.011 (3)*	
C14	0.50756 (10)	0.51032 (15)	0.43377 (5)	0.0121 (3)	
C16	0.50610 (9)	0.64963 (16)	0.45565 (5)	0.0132 (3)	
C15	0.41851 (10)	0.43285 (14)	0.43127 (4)	0.0126 (3)	
C17	0.25169 (11)	0.43452 (17)	0.45179 (5)	0.0218 (3)	
H17A	0.2435	0.3821	0.4206	0.037 (2)*	
H17B	0.2010	0.5093	0.4539	0.037 (2)*	
C18	0.24101 (12)	0.32972 (19)	0.49360 (5)	0.0272 (4)	
H18A	0.2945	0.2609	0.4932	0.037 (2)*	
H18B	0.1803	0.2770	0.4903	0.037 (2)*	
H18C	0.2412	0.3832	0.5245	0.037 (2)*	

### Atomic displacement parameters (Å^2^)

**Table:**

	*U*^11^	*U*^22^	*U*^33^	*U*^12^	*U*^13^	*U*^23^
Zn1	0.01164 (9)	0.00914 (9)	0.00874 (8)	−0.00053 (5)	0.00045 (5)	−0.00140 (5)
O1	0.0152 (5)	0.0196 (5)	0.0134 (4)	−0.0059 (4)	0.0007 (3)	−0.0042 (4)
O2	0.0143 (5)	0.0136 (5)	0.0122 (4)	−0.0016 (4)	0.0011 (3)	−0.0024 (3)
O3	0.0141 (5)	0.0166 (5)	0.0226 (5)	0.0008 (4)	0.0038 (4)	−0.0058 (4)
O4	0.0156 (5)	0.0132 (5)	0.0126 (4)	−0.0006 (4)	0.0013 (4)	−0.0028 (3)
O5	0.0387 (7)	0.0089 (5)	0.0110 (5)	0.0003 (4)	0.0024 (4)	−0.0005 (4)
N1	0.0131 (5)	0.0096 (5)	0.0095 (5)	0.0002 (4)	0.0000 (4)	−0.0002 (4)
N2	0.0121 (5)	0.0092 (5)	0.0102 (5)	−0.0003 (4)	−0.0010 (4)	−0.0007 (4)
N3	0.0143 (6)	0.0172 (6)	0.0136 (5)	−0.0040 (5)	−0.0001 (4)	−0.0026 (4)
N4	0.0218 (7)	0.0124 (6)	0.0161 (6)	0.0003 (5)	0.0043 (4)	−0.0015 (5)
C1	0.0134 (6)	0.0088 (6)	0.0092 (5)	0.0013 (5)	−0.0007 (4)	0.0015 (4)
C2	0.0163 (7)	0.0097 (6)	0.0120 (6)	−0.0008 (5)	−0.0023 (5)	−0.0005 (4)
C3	0.0138 (6)	0.0111 (6)	0.0142 (6)	−0.0024 (5)	−0.0030 (5)	0.0020 (5)
C4	0.0122 (6)	0.0136 (6)	0.0140 (6)	0.0007 (5)	0.0015 (5)	0.0016 (5)
C5	0.0153 (6)	0.0116 (6)	0.0120 (6)	0.0004 (5)	−0.0004 (5)	−0.0018 (5)
C6	0.0137 (6)	0.0086 (6)	0.0097 (5)	−0.0005 (5)	−0.0010 (4)	0.0013 (4)
C7	0.0130 (6)	0.0095 (6)	0.0101 (5)	0.0006 (5)	0.0006 (4)	0.0004 (4)
C8	0.0140 (6)	0.0113 (6)	0.0098 (6)	−0.0011 (5)	−0.0008 (5)	−0.0012 (5)
C9	0.0137 (6)	0.0095 (6)	0.0116 (5)	−0.0001 (5)	−0.0022 (5)	0.0006 (4)
C10	0.0106 (6)	0.0118 (6)	0.0126 (6)	−0.0026 (5)	−0.0014 (5)	0.0021 (5)
C11	0.0121 (6)	0.0193 (7)	0.0205 (6)	−0.0047 (5)	0.0026 (5)	−0.0045 (5)
C12	0.0253 (8)	0.0162 (7)	0.0243 (7)	−0.0028 (6)	0.0065 (6)	−0.0015 (5)
C13	0.0147 (6)	0.0103 (6)	0.0085 (5)	−0.0008 (5)	−0.0014 (5)	0.0003 (4)
C14	0.0165 (7)	0.0095 (6)	0.0105 (6)	0.0014 (5)	0.0005 (4)	−0.0012 (5)
C16	0.0156 (7)	0.0137 (7)	0.0103 (6)	0.0005 (5)	0.0021 (4)	0.0016 (5)
C15	0.0151 (7)	0.0131 (6)	0.0096 (5)	0.0024 (5)	0.0008 (5)	0.0009 (5)
C17	0.0116 (7)	0.0269 (8)	0.0268 (7)	0.0002 (6)	0.0006 (5)	−0.0103 (6)
C18	0.0210 (8)	0.0365 (9)	0.0242 (7)	−0.0136 (7)	0.0030 (6)	−0.0085 (7)

### Geometric parameters (Å, º)

**Table:**

Zn1—O4	2.0606 (9)	C3—H3	0.9500
Zn1—N2	2.0626 (11)	C4—C5	1.3831 (19)
Zn1—N1	2.0754 (11)	C4—H4	0.9500
Zn1—O2	2.1112 (9)	C5—C6	1.4016 (18)
Zn1—O5	2.1852 (11)	C5—H5	0.9500
Zn1—N3^i^	2.2317 (11)	C7—C8	1.4141 (18)
O1—C9	1.3476 (16)	C7—H7	0.9500
O1—C11	1.4555 (16)	C8—C10	1.4175 (18)
O2—C9	1.2447 (15)	C8—C9	1.4304 (18)
O3—C15	1.3440 (16)	C11—C12	1.511 (2)
O3—C17	1.4582 (18)	C11—H11A	0.9900
O4—C15	1.2455 (16)	C11—H11B	0.9900
O5—H51	0.79 (2)	C12—H12A	0.9800
O5—H52	0.83 (2)	C12—H12B	0.9800
N1—C13	1.3064 (16)	C12—H12C	0.9800
N1—C1	1.4161 (17)	C13—C14	1.4242 (18)
N2—C7	1.2970 (16)	C13—H13	0.9500
N2—C6	1.4097 (17)	C14—C16	1.4192 (19)
N3—C10	1.1493 (17)	C14—C15	1.4336 (19)
N3—Zn1^ii^	2.2317 (11)	C17—C18	1.508 (2)
N4—C16	1.157 (2)	C17—H17A	0.9900
C1—C2	1.3982 (18)	C17—H17B	0.9900
C1—C6	1.4163 (17)	C18—H18A	0.9800
C2—C3	1.3862 (19)	C18—H18B	0.9800
C2—H2	0.9500	C18—H18C	0.9800
C3—C4	1.3962 (18)		
			
O4—Zn1—N2	167.20 (4)	N2—C6—C1	116.22 (12)
O4—Zn1—N1	90.06 (4)	N2—C7—C8	125.34 (12)
N2—Zn1—N1	79.83 (4)	N2—C7—H7	117.3
O4—Zn1—O2	102.78 (4)	C8—C7—H7	117.3
N2—Zn1—O2	87.65 (4)	C7—C8—C10	115.48 (12)
N1—Zn1—O2	167.05 (4)	C7—C8—C9	124.60 (12)
O4—Zn1—O5	83.64 (4)	C10—C8—C9	119.15 (12)
N2—Zn1—O5	104.21 (4)	O2—C9—O1	121.16 (12)
N1—Zn1—O5	90.97 (4)	O2—C9—C8	126.50 (12)
O2—Zn1—O5	88.89 (4)	O1—C9—C8	112.33 (11)
O4—Zn1—N3^i^	87.06 (4)	N3—C10—C8	176.10 (14)
N2—Zn1—N3^i^	85.45 (4)	O1—C11—C12	110.65 (12)
N1—Zn1—N3^i^	91.86 (4)	O1—C11—H11A	109.5
O2—Zn1—N3^i^	90.43 (4)	C12—C11—H11A	109.5
O5—Zn1—N3^i^	170.28 (4)	O1—C11—H11B	109.5
C9—O1—C11	117.81 (10)	C12—C11—H11B	109.5
C9—O2—Zn1	125.02 (9)	H11A—C11—H11B	108.1
C15—O3—C17	117.09 (11)	C11—C12—H12A	109.5
C15—O4—Zn1	126.05 (9)	C11—C12—H12B	109.5
Zn1—O5—H51	133.8 (15)	H12A—C12—H12B	109.5
Zn1—O5—H52	110.0 (16)	C11—C12—H12C	109.5
H51—O5—H52	111 (2)	H12A—C12—H12C	109.5
C13—N1—C1	121.06 (11)	H12B—C12—H12C	109.5
C13—N1—Zn1	124.65 (9)	N1—C13—C14	125.16 (12)
C1—N1—Zn1	113.18 (8)	N1—C13—H13	117.4
C7—N2—C6	120.25 (11)	C14—C13—H13	117.4
C7—N2—Zn1	124.61 (9)	C16—C14—C13	117.16 (12)
C6—N2—Zn1	113.73 (8)	C16—C14—C15	117.38 (12)
C10—N3—Zn1^ii^	156.83 (11)	C13—C14—C15	125.44 (12)
C2—C1—N1	125.81 (11)	N4—C16—C14	179.05 (15)
C2—C1—C6	118.41 (12)	O4—C15—O3	120.33 (12)
N1—C1—C6	115.77 (12)	O4—C15—C14	126.80 (12)
C3—C2—C1	120.83 (12)	O3—C15—C14	112.87 (11)
C3—C2—H2	119.6	O3—C17—C18	111.08 (12)
C1—C2—H2	119.6	O3—C17—H17A	109.4
C2—C3—C4	120.58 (13)	C18—C17—H17A	109.4
C2—C3—H3	119.7	O3—C17—H17B	109.4
C4—C3—H3	119.7	C18—C17—H17B	109.4
C5—C4—C3	119.55 (13)	H17A—C17—H17B	108.0
C5—C4—H4	120.2	C17—C18—H18A	109.5
C3—C4—H4	120.2	C17—C18—H18B	109.5
C4—C5—C6	120.49 (12)	H18A—C18—H18B	109.5
C4—C5—H5	119.8	C17—C18—H18C	109.5
C6—C5—H5	119.8	H18A—C18—H18C	109.5
C5—C6—N2	123.66 (11)	H18B—C18—H18C	109.5
C5—C6—C1	120.06 (12)		

Symmetry codes: (i) −*x*+1, *y*+1/2, −*z*+1/2; (ii) −*x*+1, *y*−1/2, −*z*+1/2.

### Hydrogen-bond geometry (Å, º)

**Table:**

*D*—H···*A*	*D*—H	H···*A*	*D*···*A*	*D*—H···*A*
O5—H51···N4^iii^	0.79 (2)	2.21 (2)	2.9823 (17)	168 (2)
O5—H52···N4^iv^	0.83 (2)	2.12 (2)	2.9405 (16)	174 (2)

Symmetry codes: (iii) *x*, *y*−1, *z*; (iv) −*x*+1, −*y*+1, −*z*+1.
